# Reviewing Clinical Effectiveness of Active Training Strategies of Platform-Based Ankle Rehabilitation Robots

**DOI:** 10.1155/2018/2858294

**Published:** 2018-02-20

**Authors:** Xiangfeng Zeng, Guoli Zhu, Mingming Zhang, Sheng Q. Xie

**Affiliations:** ^1^School of Mechanical Science and Engineering, Huazhong University of Science and Technology, Luoyu Road 1037, Wuhan, China; ^2^Department of Mechanical Engineering, University of Auckland, Auckland 1142, New Zealand; ^3^The State Key Laboratory of Digital Manufacturing Equipment and Technology, Huazhong University of Science & Technology, Luoyu Road 1037, Wuhan, China; ^4^School of Mechanical Engineering and School of Electronic and Electrical Engineering, University of Leeds, Leeds LS2 9JT, UK

## Abstract

**Objective:**

This review aims to provide a systematical investigation of clinical effectiveness of active training strategies applied in platform-based ankle robots.

**Method:**

English-language studies published from Jan 1980 to Aug 2017 were searched from four databases using key words of “Ankle^∗^” AND “Robot^∗^” AND “Effect^∗^ OR Improv^∗^ OR Increas^∗^.” Following an initial screening, three rounds of discrimination were successively conducted based on the title, the abstract, and the full paper.

**Result:**

A total of 21 studies were selected with 311 patients involved; of them, 13 studies applied a single group while another eight studies used different groups for comparison to verify the therapeutic effect. Virtual-reality (VR) game training was applied in 19 studies, while two studies used proprioceptive neuromuscular facilitation (PNF) training.

**Conclusion:**

Active training techniques delivered by platform ankle rehabilitation robots have been demonstrated with great potential for clinical applications. Training strategies are mostly combined with one another by considering rehabilitation schemes and motion ability of ankle joints. VR game environment has been commonly used with active ankle training. Bioelectrical signals integrated with VR game training can implement intelligent identification of movement intention and assessment. These further provide the foundation for advanced interactive training strategies that can lead to enhanced training safety and confidence for patients and better treatment efficacy.

## 1. Introduction

Human ankle joints are very complex with ligaments and muscles bonded, the main function of which is to maintain the balance of human bodies when they are standing and provide a forward or backward force when they are walking [1, 2]. As one of the most fragile portions in the human body, ankle joints are easy to get injured in daily life when they experience unexpected force or suffer diseases. Stroke has been listed as one of the main reasons for ankle disability [3], with the number of stroke patients increasing yearly at a rate of approximate 795,000 in the United States. In 2013, over 7.5 million of stroke patients existed in China, with an increasing proportion of younger patients [4]. In New Zealand, there is an estimated number of 60,000 stroke patients; many of them have an abnormal gait pattern [5].

Patients with ankle disability partially or totally lose their motion ability. This can result in the lack of movements to maintain joint range of motion (ROM) simply based on their own efforts. With the elapse of time, drop foot, amyotrophy, and severe passive ankle stiffness (PAS) can be caused when without sufficient ankle rotations [6]. Traditional ankle physical therapy is delivered manually by therapists [7]. Many assistive and rehabilitation devices have been developed over the past few decades, and the emerging of robot-assisted ankle rehabilitation techniques bring a new breakthrough with great potential for clinical applications [8, 9]. Ankle robots can provide accurate and uniform rehabilitation exercises in a long-time session of the training, the difficulty of which can be adaptively modified based on real-time feedback of therapeutic effects [10]. Also, the use of robot-assisted ankle rehabilitation technique allows for real-time data collection throughout the training, to further judge the accuracy of the training [5], evaluate the biomechanical properties of ankle joints [11, 12], assess the performance of motion ability of ankle joints [10, 13], and customize future therapies [14]. Ankle robots can be classified to two kinds, one of which is wearable ankle robots applied to improve gait and locomotor ability, and the other of which is platform-based robots applied in a sitting position to enhance physical function and motion ability of ankle joints [15]. Until now, various ankle rehabilitation robots have been developed based on different kinematics modules, mechanical structures, and training strategies [16–20], where some clinical tests were conducted to evaluate their effectiveness for treating ankle injuries.

Robot-assisted rehabilitation techniques involve the delivery of both passive and active training. For passive training, subjects are always requested to keep relaxed when their ankle joints follow up rotation trajectories of robots [21]. After a period of passive training, partial function of ankle joints can be maintained and foot drop can be alleviated correspondingly [22]. For active training, subjects are required to accomplish a task within a predefined time through rotating ankle joints with assistance from robots following visual or auditory instructions [23]. Differently from passive training, active training can exercise the information transmission loop between the brains and ankle joints, and evidences demonstrate that conducting active training could achieve more improvement for patients in walking speed, motor control, and gait patterning [21]. However, a comprehensive review of active training strategies applied with platform ankle robots is lacking. This paper aims to provide a systematical investigation of the clinical effectiveness of active training strategies applied in platform-based ankle robots.

## 2. Methods

### 2.1. Search Strategy

Only English-language studies published from January 1980 to August 2017 were searched in the following four databases: Scopus, Web of Science, ScienceDirect, and Embase. Key words for searching were set up as “Ankle^∗^” AND “Robot^∗^” AND “Effect^∗^ OR Improv^∗^ OR Increas^∗^”.

**Figure 1:**
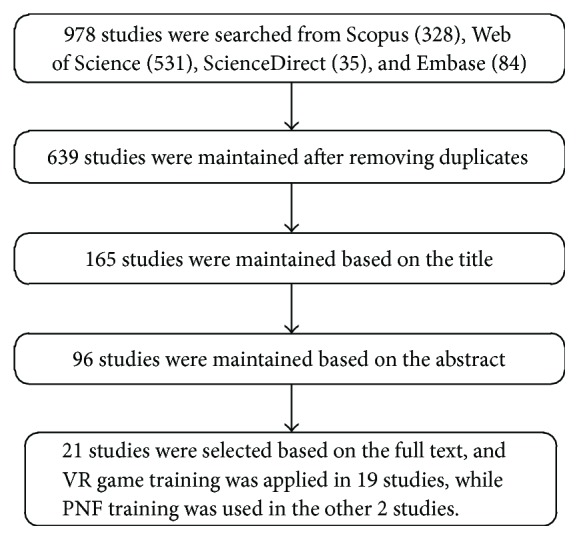
Flow diagram of selection process for final review.

A total of 639 studies were obtained after removing 339 duplicates from the initial search result, and further discrimination was conducted successively based on the title, the abstract, and the full text of the studies. The selection process is described in Figure 1, where the inclusion and exclusion criteria are described next.

### 2.2. Inclusion and Exclusion Criteria

This study aims to provide a systematical investigation of clinical effectiveness of active training strategies applied in platform-based ankle robots. Inclusion criteria are (1) studies involving platform-based ankle robots and (2) studies involving clinical research of ankle rehabilitation training. Exclusion criteria were (1) studies whose training was conducted not in a seated position, (2) studies whose purpose was to propose and verify novel technologies and algorithms, (3) studies whose intervention protocol simply contained the passive training, (4) studies whose purpose was to verify the feasibility of novel robots, (5) studies involving only one subject, (6) conference papers whose content were merged into a peer-reviewed journal one, and (7) studies whose purpose was to analyze the biomechanics of ankle joints. Only English-language studies published in peer-reviewed journals or conference papers were included.

## 3. Result

After excluding studies involving training conducted not in a seated position [1, 24–29], proposition and verification of novel technologies and algorithms [22, 30–37], single passive training [5, 38, 39], proposition and verification of novel robots [8, 16–19, 40–45], single subject [46], conference papers merged into peer-reviewed journals [47–49], and analyzing the biomechanics of ankle joints [11, 12, 50, 51], a total of 21 studies were finally selected with 311 patients involved; of them, 13 studies applied a single group while another eight studies used different groups for comparison to verify the therapeutic effect. Virtual-reality (VR) game training was applied in 19 studies, while two studies used proprioceptive neuromuscular facilitation (PNF) training, as shown in Table 1.

## 4. Discussion

The purpose of this review is to provide a systematical investigation of clinical effectiveness of active training strategies applied in platform-based ankle robots. This review will be discussed from two main aspects: achievements of clinical research and training strategies.

### 4.1. Achievements of Clinical Research

VR game training has been demonstrated to be a good selection for ankle rehabilitation therapies, even a single session of VR game training can greatly improve motor control ability of paretic ankle joints, to be more effective if integrated with a locomotor treadmill, and to be more beneficial to subjects with moderate and mild gait speed impairments. Mirelman et al. [13] arranged eighteen subjects with chronic hemiparesis equally to a robot VR group and a robot group. Subjects in the robot VR group executed ankle training with VR feedback, and subjects in the robot group conducted the same training simply with instructions informed by a therapist every 30 seconds. The robot VR group achieved better performance in walking than the robot group did. Forrester et al. [52] suggested that VR game training can be a valuable supplement to locomotor therapies, based on the observation that patients with chronic stroke not only improved motor ability of paretic ankle joints realizing faster and smoother movements but also significantly increased walking velocity after a 6-week 18-session training. Waldman et al. [53] recruited 23 stroke survivors to a robot group and a control group. The robot group conducted VR game training in hospital, and the control group conducted the similar training manually following verbal and written instructions at home. It was observed that the robot group achieved more significant improvement in motor function and mobility than the control group did after an 18-session clinical trial. Roy et al. [54] observed that immediately following and 48 hours after a single session of VR game training, motor control of paretic ankle joints was improved with significant positive changes in success, speed, and smoothness of targeted ankle movements but not for nondisabled ankles. Forrester et al. [55] concluded that VR game training was more effective when it was integrated with a locomotor treadmill, based on the observation that the TMR (treadmill robotic) group achieved better improvement in gait biomechanics and paretic ankle function than the SRT (seated robotic training) group did after an 18-session training. Chang et al. [56] arranged a 6-week 18-session VR game training for 29 subjects with hemiparesis, who were categorized to low, moderate, and high-function groups according to gait speed performance levels. It was concluded that the therapeutic effect of conducting VR game training was better for subjects with better gait speed performance, based on the observation that the high-function group recovered their comfortable gait speed to the normal and the moderate-function group also received a positive change in gait speed but the low-function group did not get an obvious alteration in gait speed. Forrester et al. [57] summarized its clinical effectiveness of the anklebot for stroke rehabilitation by reviewing pilot studies using seated visuomotor anklebot training with chronic patients, along with results from initial efforts to evaluate the anklebot's utility as a clinical tool for assessing intrinsic ankle stiffness.

VR game training following up the clinical protocol has similar effect as the one under the lab protocol, which has been successfully and efficiently applied to subjects in the early subacute phase of stroke, to children with CP, to patients with multiple sclerosis (MS), and to patients in bed. Sukal-Moulton et al. [58] investigated the therapeutic effect of a clinic cohort, the intervention of which was designed by therapists, through comparing with an existing research cohort. It was suggested that VR game training was feasible for clinical applications, based on the observation that the clinic cohort achieved equivalent clinical improvements to those attained from the research cohort. Forrester et al. [21] divided 34 patients in the early subacute phase of stroke to a robot group and a stretching group. The robot group conducted VR game training while the stretching group received manual passive stretching. It was observed that the robot group achieved more improvement in walking speed, motor control, and gait patterning than the stretching group did. Wu et al. [59] arranged 12 children with spastic CP to conduct VR game training and concluded that VR game training was beneficial for children with CP, based on the improvement in ankle biomechanical properties, performance of motor control, and functional capability in balance and mobility after the training. Krebs et al. [60] concluded that VR game training on pediAnklebot could provide better therapeutic effect on ankle rehabilitation among children with CP, based on the improvement of the physical function of ankle joints in pointing abilities and gait speed after the training. Lee et al. [61] concluded that VR game training could provide subjects with MS better therapeutic effect on sensorimotor functions of lower limbs, based on great improvements of rotation abilities and sensory functions of ankle joints after the training. Ren et al. [62] demonstrated that in-bed VR game training met clinical requirements based on the significant improvement in active and passive biomechanical properties of ankle joints.

The performance of VR game training is directly mapped to the motion ability of ankle joints, which can be improved in explicit motor learning and implicit motor learning, especially if targets in VR games are progressive. Burdea et al. [10] arranged three pediatric subjects with CP to conduct a 36-session VR game training with the difficulty gradually increased in the process. It was concluded that the performance of playing the game was mapped to the physical improvements evaluated clinically in ankle strength, gait kinematics, and speed. Michmizos et al. [63] arranged three children with CP to conduct a 9-session VR game training as quickly and accurately as possible. It was concluded that participants obtained significant improvement in explicit motor learning assessed with less jerky, better controlled, and increased speed of movements and implicit motor learning evaluated by the reduction of the average reaction time (RT). Roy et al. [2] arranged eight subjects with chronic stroke to conduct an 18-session VR game training, which could inspire participants' motivation by integrating the function of performance-based progression. It was found that VR game training with progressive targets significantly decreased PAS of paretic ankle joints, even to the normal range of dorsiflexion.

Neural plasticity at the cortical can be induced by robot-assisted stretching triggered by motor imagination and be accelerated to form when rewards are integrated with VR game training. Xu et al. [64] arranged nine healthy subjects to conduct robot-assisted passive stretching which were triggered by motion intention extracted from electroencephalograph (EEG) signals. The neural plasticity at the cortical was induced based on the observation that the size of the motor-evoked potential (MEP) elicited by transcranial magnetic stimulation (TMS) significantly increased immediately following and 30 minutes after the training. Goodman et al. [23] suggested that when subjects with chronic hemiparetic stroke conducted VR game training, rewards integrated with the performance could accelerate activity-dependent brain plasticity to improve motor control ability of ankle joints.

VR game training with teleassistance is effective, and its therapeutic effect will not be significantly affected by whether therapists are on-site or online. Chen et al. [65] arranged 23 patients with CP to conduct an 18-session VR game training at home with remote technical support and communication from therapists. It was found that VR game training with teleassistance is not only convenient and economical but also effective based on significant improvement in passive and active joint ROM, muscle strength, spasticity, and balance after the training at home. Chen et al. [14] divided 41 children with CP to a laboratory-based group and a home-based group to conduct the same VR game training. This also suggested that VR game training is feasible to be conducted at home since both groups received similar benefits after the training. Deutsch et al. [66] demonstrated that therapeutic effects of injured ankles were not significantly different for chronic stroke patients with a 12-session VR game training, no matter whether therapists were in the same room with patients or appeared in front of patients remotely.

Robot-assisted PNF stretching is effective for injured ankles. Zhou et al. [67] arranged 5 patients with chronic stroke to conduct an 18-session PNF stretching. It was concluded that robot-assisted PNF stretching was an effective therapy method to rehabilitate ankle joints with contracture and spasticity, based on the improvement of ROM and the resistant torque after the PNF training. Zhou et al. [68] draw a further conclusion that robot-assisted PNF stretching was effective in alleviating spasticity of lower limbs and improving motor function.

### 4.2. Training Strategies

One basic function of training strategies applied with ankle robots is to provide feedback for subjects through transferring real-time parameters of ankle training to visual, auditory, and haptic information. This can provide an intelligent stretching through comprehensively analyzing motion ability of ankle joints and rehabilitation plans. Bioelectrical signal can also be applied to control the ankle training. In this section, training strategies will be discussed from three aspects: robot-assisted feedback, intelligent and interactive training strategies, and the use of bioelectrical signals.

#### 4.2.1. Robot-Assisted Feedback

A robot can not only provide real-time visual and auditory feedback, but also set up emotional and haptic feedback to exercise motion ability of ankle joints. VR game training can provide visual and auditory feedback [2, 21, 23, 52–55, 63, 66]. It usually starts with a target emerging suddenly at the VR circumstance, the cursor of which will be manipulated to achieve the target through actively rotating ankle joints [53, 59, 60]. When VR game training is conducted, visual or auditory feedback provided by robots enables subjects to acquire real-time performance of the training in rate of progress, speed, number of jerk, smoothness of movement track, whether the cursor achieves goals, scoring, and so on [63]. By analyzing those information, subjects can efficiently allocate their energy to rotate ankle joints to achieve targets. After several loops of information transferring and ankle joint movements, one round of VR game training will be accomplished when the target is reached by the cursor [58]. Without feedback provided from VR circumstance, subjects will not be able to completely handle the training simply based on their own qualitative analysis and judgment; even information is provided by physical therapists [13]. VR game training has been demonstrated to achieve better performance than similar robot-assisted training without VR feedback [13]. In general, VR game training can inspire the enthusiasm of subjects to accomplish the training through transferring boring ankle training to interesting games with visual and auditory feedback. To some extent, VR game training improves the therapeutic effect.

Rewards can provide emotional feedback for subjects to inspire their enthusiasm to accomplish the goals in the ankle training. Rewards are divided to immediate rewards which should be achieved within a session and long-term rewards which can be achieved throughout the whole rehabilitation program [23]. Rewards integrated with VR game training are regarded as another parameter both for patients and physical therapists to evaluate therapeutic effects. Rewards represent a comprehensive assessment result through analyzing the performance of all evaluation parameters, such as the number of jerk, the smoothness of movement track, the spending time, speed, and the number of success. Thus, the performance of the training can be judged directly by patients based on the level of rewards achieved. Rewards are in accordance with a universal value in the living that better performance can lead to better rewards. In this circumstance, subjects always have strong motivations to earn more rewards since better performance will result in higher probability to recover from their disability. Studies suggested that rewards associated with the performance when subjects conducted VR game training could accelerate activity-dependent brain plasticity to improve their motor control ability of ankle joints [23].

Haptic effects simulate the feeling of subjects assuming they are being in the VR game scene. Haptic effects integrated with VR game training are divided to low-level haptic and task-level effects. Low-level haptic effects are applied to support subjects as a compensation of the gap between the actual position and the desired one through modifying properties of robots, such as impedance gains and proportional gains. Task-level haptic effects are utilized to be a disturbance by modifying suddenly and fiercely the position of rotating platforms [69]. Haptic feedback can be selectively applied in the ankle training. A task-level haptic effect was applied in [10] as one of the parameters to modify the difficulty of training in the Rutgers Ankle CP by altering its intensity. Haptic feedback was selectively applied to support subjects [13], where the robot VR group was with the support of the low-level force feedback and the task-level haptic feedback, but the robot group was assisted only with the low-level force feedback. Compared with exercises without haptic effects, the same training with task-level haptic effects achieved significant improvements in accuracy, mechanical power of impaired ankles, repetitions of ankle movements, duration of training, and efficiency of training time [66]. In general, haptic feedback cooperates with visual and auditory feedback originating from the VR game, which enables subjects to better control the VR game training. Disturbance accompanying with task-level haptic effects can increase the difficulty for participants to control and accomplish VR training tasks.

When performing hold-reflex PNF stretching, injured ankle joints are stretched passively by robots to arrive and stay at the extreme dorsiflexion position, where subjects are requested to actively conduct plantarflexion with real-time recording of electromyography (EMG) value being in the range of 40% to 60% MVC during a prescribed time, and then ankle joints are rotated back to their neutral position [67]. Further relaxation of soleus muscles can be resulted from active contraction in PNF stretching, repetitions of which can enable subjects to contract and relax their ankle joints [68]. Compared with manual PNF training, subjects can easily control the variation range of voluntary contractions in a predefined range when robot-assisted PNF stretching is conducted. Differently from passive stretching, active participation and force control is the key to conduct the PNF stretching. Its therapeutic effect significantly depends on participants themselves.

#### 4.2.2. Intelligent and Interactive Training Strategies

Intelligent passive training is a robot-assisted passive ankle stretching widely applied together with active ankle training, because of its safety and efficiency. The rotating speed of intelligent passive stretching can be set to be inversely proportional to the resistance torque of ankle joints [22]. When ankle joints rotate toward their limitation of ROM, the related muscle tendon will be stretched slowly because increased resistance torque of ankle joints gradually slows down the robot [70]. When a predefined maximum resistance torque value is achieved, the ankle joint is at that position for a period of time to allow stress relaxation and then is stretched back to its neutral position [53]. When conducting intelligent passive stretching, subjects are requested to feel relaxed with ankle joints being stretched to follow up predefined motion trajectories of the robot and without resistance force generated from their ankle joints [22]. In the clinical research, intelligent passive stretching is usually regarded as either a process of warming up to energize impaired ankle joints or a process of cooling down to eliminate the tension of ankle joints at the end of the training [14, 49, 53, 59, 65]. In general, intelligent passive stretching can avoid accidental injuries to ankle joints, because of its inverse proportion between the rotating speed and the resistance torque of ankle joints.

The function of the “assist-as-needed” strategy can support subjects to achieve the targets through providing necessary assistance, which can be computed based on the location gap between the predefined target and the cursor manipulated by the ankle joint, when subjects cannot activate the rotation of ankle joints within a predefined time or cannot reach the targets in time [2, 52, 54]. The provided assistance from robots can be adjusted through modifying the stiffness parameter of the controller [23]. The function of “assist as needed” will not be activated if robots cannot detect the rotation force of ankle joints [55, 63]. In general, the function of the “assist-as-needed” control strategy enlarges the application of VR game training to subjects who cannot control VR games based on their own motion ability of ankle joints. When the rotation force of ankle joints is detected by sensors built in robots, subjects can conduct VR game training successfully with necessary assistance. The confidence of subjects will be enhanced greatly when they can smoothly control the whole processes and finally reach the targets with a higher success rate.

Active training can be assisted active movements, assistance torques of which are provided by ankle robots to assist subjects to accomplish targets designed in VR game training, or resisted active movements, resistance torques of which are supplied by ankle robots to increase the difficulty of conducting the VR training game [14, 49, 53, 58, 59, 61, 65]. Compared with the “assist as needed,” assisted active movements provide a similar function but without considering whether subjects can achieve the targets simply through their own effort. It can assist subjects to complete the VR game training easily; meanwhile, their confidence to succeed in the next training is enhanced. Resisted active movement mode allows subjects to achieve targets slightly beyond their ability, which requires them to pay more attention and effort to accomplish VR game training. In general, both active movement modes are mostly combined together in a clinical session where assisted active movements are conducted in advance as a warm-up exercise, while resisted active movements mainly focus on breakthrough of motion ability of ankle joints.

The strategy of “easy to difficult” is applied on robots for subjects to maintain enthusiasm and avoid frustration through decreasing assistance strength in a session [2, 10, 13, 23, 52, 54, 55, 60, 63]. First is that subjects can quickly be familiar with processes and regulations of the training since stronger assistance enables them to spend only a small portion of time and energy to achieve the goals. Second, the participants can gradually build confidence in the motion ability of ankle joints, which can inspire their enthusiasm to successfully achieve goals in the subsequent training with less assistance provided by the robot. Finally, they can spare no effort to achieve goals with minimum assistance based on accumulation of familiarity, technique, and confidence during the previous training stages. In general, the strategy of “easy to difficult” can assist subjects to gradually build confidence in the ankle training and finally accomplish the training little beyond their motion ability of ankle joints.

The strategy of “performance-based progression” is another kind of intelligent training realized by evaluating the online performance to decide the parameters of the next training. When an ankle training program comprises a series of sessions, the performance achieved by subjects in each session can be different; therefore, adjusting parameters of the training in the next session according to the performance is essential. The strategy of “performance-based progression” applied in [2] increased target locations by 10% in weeks 3-4 and added frequency of target presentation by 0.06 Hz in weeks 5-6 when the success rate without robotic assistance was sustained at least 80%. Therefore, the concept of “performance-based progression” can be regarded as a kind of training strategy that challenges the participants over time by increasing the training difficulty with better performance [55].

#### 4.2.3. The Use of Bioelectrical Signals

Bioelectrical signals applied in robot-assisted ankle training mainly comprise EMG and EEG. EMG signals are originated from muscle activation and can reflect electrical activities of skeletal muscles by detecting their potentials generated by muscle cells [11]. EEG signals are recorded over a period of time from multiple electrodes placed on the scalp to display spontaneous electrical activity within the brains [71].

EMG signals generated by performing MVC can be used to indicate the motion ability of ankle joints [64, 67]. During the testing run in [64], EMG signals were applied to ensure there will not be any visible EMG activity involved when subjects conducted motor imagery. During stretch reflex measurements in [64], EMG signals were applied as an indicator for subjects at all times to maintain a 5% MVC EMG level in their right tibialis anterior (TA) when they were being interfered with the plantarflexion perturbation imposed on the ankle joints. EMG signals were utilized in [67] to confirm whether the soleus is the main source of the resistance torque generated by ankle joints when subjects were conducting dorsiflexion. EMG signals were also used in [67] as real-time feedback for subjects to maintain the soleus EMG value in the range of 40% to 60% MVC for 15 seconds. EMG signals can be also used to predict and display the force orientation of ankle joints through recording electrical activities of targeted muscles. They have been utilized in [2] to prove the assumption of zero voluntary contribution from both gastrocnemius (GAS) and TA muscles during the passive stretching. In general, this kind of application of EMG signals is an auxiliary tool for biomechanical assessment purposes.

The EEG signals have two applications when integrated with robot-assisted ankle rehabilitation training, one of which is to analyze the potential distribution of human brains, the other of which is to extract the motion intention of subjects as a switch signal to trigger the robot-assisted stretching. EEG signals were applied in [23] to evaluate whether task-related training could produce neurophysiologic adaptations related to motor learning, based on the comparison of EEG signals measured at the first training day and the last training day. Although not applied on patients, EEG signals were utilized in [64] to detect motion intention of subjects to trigger the robot to conduct passive dorsiflexion stretching through analyzing movement-related cortical potential (MRCP). In brief, one application is to make the rehabilitation training to have a similar human motion mechanism by which movement instructions come from the brains. Another application enables therapists to know the modification of potential distribution in human brains after ankle training, and then they can judge the therapeutic effect based on an assessment protocol.

### 4.3. Ideal Training Strategies for Ankle Rehabilitation

Training strategies cannot be identical for all patients; therefore, ideal training strategies should be tailored according to actual rehabilitation requirement and specific motion ability of ankle joints. A VR-based training environment has been used in most studies included in this review except [67, 68]. This strategy can not only provide visual and auditory feedback but also inspire the enthusiasm of patients to continuously conduct ankle training. The application of bioelectrical signals can be a good supplement of VR game training because they allow intelligent assessment of joint function and motion ability. Elements of intelligent stretching can be selectively applied to VR game training by considering rehabilitation requirement and motion ability of ankle joints. For instance, for patients whose ankle motion ability cannot accomplish the training with their own effort, integrating the strategy of “assist as needed” to the VR game training can support subjects to finish the training with active participation. Other strategies, such as warming up, cooling down, and challenge, can be well combined with VR training games for enhanced efficacy.

### 4.4. Limitations

An attempt was made to make sure that all published clinical studies of active ankle training strategies applied in platform-based robots were reviewed. In this review, we identified “ankle” as one of the key words, but some publications with the key term “foot,” “lower extremity,” or “lower limb” possibly representing “ankle” may result in an incomplete search. Following up our searching strategies, studies which aimed to verify the feasibility of novel robots were excluded, but their training strategies may be valuable and original. Non-English studies with related content may have been left out.

## 5. Conclusion

Active training techniques delivered by platform ankle rehabilitation robots have been widely researched over the world, and their therapeutic effects have been demonstrated with great potential for clinical applications. Training strategies are mostly combined with one another by comprehensively considering rehabilitation schemes and motion ability of ankle joints. The VR game environment has been commonly used with active ankle training since it allows for visual and auditory feedback and encourages active participation. Bioelectrical signals integrated with VR game training can implement intelligent identification of movement intention and assessment. These further provide the foundation for advanced interactive training strategies that can lead to enhanced training safety and confidence for patients and better treatment efficacy. Specific active training strategies should be customized on a patient-specific basis depending on the ankle disability level.

## Figures and Tables

**Table 1:** Summary of clinical researches of active training applied in platform-based ankle robots.

Study	Subjects (*n*)	Subject characteristics	Subject age (yr)	Group	Course of training	Robot	Control strategies	Researching achievement
Deutsch et al. [66]	6	Subjects with chronic stroke	Not stated	Single group	4 weeks 12 sessions	Rutgers Ankle	VR game training Haptic effects Telerehabilitation	Therapeutic effects of injured ankles were not significantly different when therapists were in the same room with patients or appeared in front of patients remotely by a webcam.
Mirelman et al. [13]	18	Subjects with chronic hemiparesis	41–75	Robot VR group (9) Robot group (9)	4 weeks 12 sessions	Rutgers Ankle	VR game training Easy to difficult Haptic effects	Training integrated with VR games were a better selection for ankle rehabilitation therapies.
Burdea et al. [10]	3	Subjects with CP	7–12	Single group	12 weeks 36 sessions	Rutgers Ankle CP	VR game training Easy to difficult Haptic effects	Performance of playing game was mapped to the physical improvement evaluated clinically in ankle strength, gait kinematics, and speed.
Forrester et al. [52]	8	Subjects with chronic stroke	43–75	Single group	6 weeks 18 sessions	Anklebot	VR game training Easy to difficult Assist as needed	Robotic feedback training would be a valuable supplement to locomotor therapies.
Roy et al. [54]	14	Healthy subjects Subjects with chronic stroke	49–64 53–74	Control group (7) Stroke group (7)	1 session	Anklebot	VR game training Easy to difficult Assist as needed	Firstly observed that immediately following and 48 hours after a single session of anklebot training, motor control of paretic ankles were improved but not for nondisabled ankles.
Roy et al. [2]	8	The same subjects as those in [52]	43–75	Single group	6 weeks 18 sessions	Anklebot	VR game training Easy to difficult Assist as needed Performance-based progression Application of EMG	Anklebot training with progressive targets significantly decreased PAS of paretic ankles, even to the normal range in dorsiflexion direction. Furthermore, increased compliance of paretic ankles would result in improvement in unassisted overground walking.
Goodman et al. [23]	10	Subjects with chronic hemiparetic stroke	42–82	HR group(5) LR group(5)	3 weeks 9 sessions	Anklebot	VR game training Easy to difficult Assist as needed Application of EEG Rewards	Rewards integrated with performance of subjects conducting anklebot training could accelerate activity-dependent brain plasticity to improve motor control.
Forrester et al. [21]	34	Subjects with hemiparetic stroke	57–66	Robot group (18) Stretching group (16)	10 sessions	Anklebot	VR game training Assist as needed Manual stretching	Robot group achieved more improvement in walking speed, motor control, and gait patterning than stretching group.
Forrester et al. [55]	26	Subjects with chronic hemiparetic gait	53–63	SRT group (12) TMR group (14)	6 weeks 18 sessions	Anklebot Treadmill	VR game training Easy to difficult Assist as needed Performance based progression	Anklebot therapy would be more effective if integrated with locomotor treadmill.
Michmizos et al. [63]^∗^	3	Subjects with CP or lesion of the common peroneal nerve	9	Single group in clinic	At least 3 weeks 9 sessions	PediAnklebot	VR game training Easy to difficult Assist as needed	Subjects obtained significant improvement of explicit motor learning assessed with less jerky, better controlled, and increased speed of movements and implicit motor learning evaluated by the reduction of the average RT (reaction time).
Krebs et al. [60]	4	Subjects with CP	7–11	Single group	6 weeks 12 sessions	PediAnklebot	VR game training Easy to difficult Assist as needed	PediAnklebot could provide better therapeutic effect on ankle rehabilitation through harnessing plasticity among children with CP.
Wu et al. [59]	12	Subjects with spastic CP	5–10	Single group	6 weeks 18 sessions	Portable rehabilitation robot	I-passive stretching VR game training A-active movement R-active movement	Active rehabilitation training combined with passive stretching was beneficial for children with CP.
Waldman et al. [53]	23	Subjects with poststroke	43–60	Robot group (11) Control group (12)	6 weeks 18 sessions	Portable rehabilitation robot	I-passive stretching VR game training A-active movement R-active movement	Robotic rehabilitation training should be a beneficial supplement to rehabilitation programs.
Sukal-Moulton et al. [58]	28	Subjects with CP	5–12	Single group	6 weeks 12 sessions	IntelliStretch rehabilitation robot	Passive stretching VR game training A-active movement R-active movement	Rehabilitation training combining active movement and passive stretching together was feasible in clinic application.
Chen et al. [65]	23	Subjects with CP	5–17	Single group	6 weeks 18 sessions	Portable rehabilitation robot with TELE	I-passive stretching VR game training A-active movement Telerehabilitation	Robotic rehabilitation training with teleassistance was not only convenient and economical but also effective for patients.
Ren et al. [62]	10	Subjects with acute poststroke	38–71	Single group	3 weeks 12 sessions	An in-bed wearable robotic device	I-passive stretching VR game training A-active movement R-active movement	The in-bed active movement training combined with passive stretching met clinic requirements and could improve motor ability of ankle joints.
Chen et al. [14]	41	Subjects with CP	5–17	Home-based group (23) Lab-based group (18)	6 weeks 18 sessions	A portable rehabilitation robot	I-passive stretching VR game training A-active movement R-active movement	Ankle rehabilitation training simply with audiovisual communication available from research engineers was feasible to be conducted at home.
Lee et al. [61]	6	Subjects with multiple sclerosis	44–66	Single group	6 weeks 18 sessions	IntelliStretch rehabilitation robot	Passive stretching VR game training A-active movement R-active movement	Ankle rehabilitation training could provide subjects with MS better therapeutic effect on sensorimotor functions of lower limbs.
Zhou et al. [67]^∗^	5	Subjects with chronic stroke	56–77	Single group	6 weeks 18 sessions	PNF assisted PKU-RARS	PNF stretching Passive stretching Application of EMG	Robot-assisted PNF stretching was an effective therapy method to rehabilitate ankles with contracture and spasticity.
Zhou et al. [68]	7	Subjects with poststroke	41–79	Single group	3 months 36 sessions	PNF assisted PKU-RARS	PNF stretching Passive stretching Application of EMG	Robot-assisted PNF stretching was significantly effective in alleviating spasticity of lower limb and improving its motor function.
Chang et al. [56]	29	Subjects with hemiparesis after stroke	18–81	LF group (9) MF group (11) HF group (9)	6 weeks 18 sessions	Anklebot	VR game training Assist as needed	Robot-assisted ankle training was more beneficial to moderate and mild gait speed impairments.

A-active moment = assisted active movement; R-active movement = resisted active movement; PNF = proprioceptive neuromuscular facilitation; I-passive stretching = intelligent passive stretching; VR = virtual reality; CP = cerebral palsy; EEG = electroencephalograph; EMG = electromyography; LF group = low-function group; MF group = moderate-function group; HF group = high-function group. ^∗^They have another group of subjects who applied to verify the function of the technology or system.
